# Time-Resolved Influences of Functional DAT1 and COMT Variants on Visual Perception and Post-Processing

**DOI:** 10.1371/journal.pone.0041552

**Published:** 2012-07-23

**Authors:** Stephan Bender, Thomas Rellum, Christine Freitag, Franz Resch, Marcella Rietschel, Jens Treutlein, Christine Jennen-Steinmetz, Daniel Brandeis, Tobias Banaschewski, Manfred Laucht

**Affiliations:** 1 Section for Clinical Neurophysiology and Multimodal Neuroimaging, Child and Adolescent Psychiatric Department, Technical University Dresden, Dresden, Germany; 2 Department of Child and Adolescent Psychiatry, Psychosomatics and Psychotherapy, Johann-Wolfgang-Goethe University, Frankfurt/Main, Germany; 3 Department of Child and Adolescent Psychiatry and Psychotherapy, Heidelberg University Hospital, Heidelberg, Germany; 4 Department of Child and Adolescent Psychiatry and Psychotherapy, Central Institute of Mental Health, Medical Faculty Mannheim/Heidelberg University, Heidelbert, Germany; 5 Department of Genetic Epidemiology in Psychiatry, Central Institute of Mental Health, Medical Faculty Mannheim/Heidelberg University, Heidelberg, Germany; 6 Biostatistics Department, Central Institute of Mental Health, Medical Faculty Mannheim/Heidelberg University, Heidelberg, Germany; 7 Department of Child and Adolescent Psychiatry, University of Zürich, Zürich, Switzerland; 8 Center for Integrative Human Physiology, University of Zürich, Zürich, Switzerland; 9 Department of Psychology, Division of Clinical Psychology, University of Potsdam, Potsdam, Germany; University of Wuerzburg, Germany

## Abstract

**Background:**

Dopamine plays an important role in orienting and the regulation of selective attention to relevant stimulus characteristics. Thus, we examined the influences of functional variants related to dopamine inactivation in the dopamine transporter (*DAT1*) and catechol-O-methyltransferase genes (*COMT*) on the time-course of visual processing in a contingent negative variation (CNV) task.

**Methods:**

64-channel EEG recordings were obtained from 195 healthy adolescents of a community-based sample during a continuous performance task (A-X version). Early and late CNV as well as preceding visual evoked potential components were assessed.

**Results:**

Significant additive main effects of *DAT1* and *COMT* on the occipito-temporal early CNV were observed. In addition, there was a trend towards an interaction between the two polymorphisms. Source analysis showed early CNV generators in the ventral visual stream and in frontal regions. There was a strong negative correlation between occipito-temporal visual post-processing and the frontal early CNV component. The early CNV time interval 500–1000 ms after the visual cue was specifically affected while the preceding visual perception stages were not influenced.

**Conclusions:**

Late visual potentials allow the genomic imaging of dopamine inactivation effects on visual post-processing. The same specific time-interval has been found to be affected by *DAT1* and *COMT* during motor post-processing but not motor preparation. We propose the hypothesis that similar dopaminergic mechanisms modulate working memory encoding in both the visual and motor and perhaps other systems.

## Introduction

Event-related potentials offer the possibility to examine the influence of genes on cognitive processes with a high time resolution. In visual processing, various stages can be described during perception, ranging from the spatial analysis of a visual stimulus during the P1 and N1 [1] to direct visual attention to the subsequent fine-grained analysis of pattern details during the N2-complex [2]. Visual perception is followed by P3 components indexing a working memory update and stimulus evaluation [3], [4]. Moreover, in paradigms which require a further memory maintenance of the stimulus, memory encoding takes place during the early contingent negative variation (CNV) [5], [6] during visual post-processing, i.e. during stimulus-dependent visual processing after stimulus offset and the completion of the initial perception. A CNV is evoked by delayed match-to-sample tasks but also by simple cued reaction time tasks, when a cue predicts a subsequent target stimulus. Previous studies have established that CNV is composed of components which depend upon the cue S1 (and also occur in a very similar way after single stimuli of the same modality) or which depend on the target stimulus S2 (stimulus anticipation and response preparation) [7]. The early CNV is conditional of S1 and is thought to represent an orienting reaction [7] which is strongly influenced by the modality of the cue [8] but not the modality of the target stimulus or whether a motor response is required [7]. The early CNV peaks early after S1 and decays to baseline towards S2, while late CNV related to response preparation and stimulus anticipation peaks right before S2 [9]. Source analysis of a visual CNV paradigm has yielded important generators of early CNV both in the frontal cortex (anterior cingulate cortex) as well as in the occipito-temporal visual cortex [10]. While frontal potentials have been associated with orienting and recruitment of resources for task performance [9], [10], modality-specific encoding in visual areas during the same time interval has been proposed to represent an important short-term memory buffer [5], [6]. This late negativity over occipito-temporal areas occurs about half a second after single visual, but not auditory or somatosensory stimuli [11], [12]. Both timing and topography thus distinguish this postprocessing component from components related to cognitive or motor preparation. The prolonged modality-dependent processing despite the short stimulus duration of 150 ms (referred to here as ‘post-processing’ [11], [12], [14], [15], [16]) is particularly important because the effective selection of relevant aspects of a stimulus often depends on the completion of stimulus perception and evaluation. In the case of short-lasting stimuli, the selective working memory encoding of relevant stimulus characteristics must thus take place after the end of the perceptual stimulation, i.e. during the post-processing interval. Automatic attention capture by single stimuli is sufficient to evoke this visual post-processing and working memory encoding even without an explicit memory task [11], [12]. This equivalent of early CNV in paired stimulus tasks has been termed N700 when evoked by single stimuli [11], [12]. Post-processing which exceeds the stimulus duration or initial stimulus perception (respectively movement execution) occurs over modality-dependent areas following short visual stimuli [6], [13], movements [14], [15], somatosensory stimuli [16], and auditory stimuli [5], [11]. In contrast to these modality-dependent activations during early CNV, a frontal negativity which adds to the modality dependent activation has been found for all sensory modalities [11], [12] and may therefore reflect a supramodal response. It has been related to an orienting response [9] because it can habituate rapidly [17].

Dopamine modulates the neuronal signal-to-noise ratio in order to focus prefrontal cortical resources [18] and plays an important role in focusing attention on the relevant stimulus characteristics [19]. The duration of dopaminergic action is limited by dopamine inactivation, i.e. mainly through methylation by catechol-O-methyltransferase (COMT) in the prefrontal cortex [20] and reuptake via the dopamine transporter (DAT1) in the striatum [21]. We recently demonstrated that DAT1 and COMT influenced specifically motor post-processing (i.e. motor N700) while pre-movement potentials were unaffected [22]. Because we found that N700 occurs across different modalities with a comparable time-course [11], here we investigated the effects of three functional polymorphisms in the *COMT* and *DAT1* genes on time resolved visual processing. The analyses ranged from initial stimulus perception (P1, N2) to stimulus post-processing after the offset of the visual stimulus (early CNV).

We examined a functional *COMT* polymorphism, characterized by the substitution of valine for methionine at codon 158 [23], resulting in less enzyme activity and higher extracellular dopamine levels [24]. With respect to DAT1, the 10-repeat allele of a variable number tandem repeat (VNTR) polymorphism in the 3′-untranslated-region of *DAT1* and the 6-repeat allele of a VNTR in intron 8 have been found to lead to greater DAT1 expression [25] and reduced striatal dopamine levels. The co-occurrence of both DAT1-expression increasing VNTRs, the 6R–10R haplotype, has been described to strengthen this effect [26], [27]. However, the controversy about whether the 10-repeat allele leads to greater or lower DAT expression [28] is not yet finally resolved. Functional responses in the striatum (e.g. reward cue-related BOLD responses or dopamine release) have been found to be larger for carriers of the 9R-allele in some but not all studies [29], [30]. Developmental aspects may change gene expression, as, in children, the 6R–10R-allele has been described to be a risk factor for ADHD, while in adults the 6R–9R allele was associated with ADHD [31], [32], pointing towards a differential decay of dopamine transporter expression with development [28], i.e. a steeper age-related decrease of dopamine binding capacity for non 6R–10R carriers. Though this adds some caveats to the interpretation of molecular mechanisms behind influences of *DAT1* genotype on EEG or neuroimaging data, there is a wide agreement that this *DAT1* polymorphism is functional. Thus, here we examined the *DAT1* haplotype and its interaction with the *COMT* Val^158^Met polymorphism in relation to visual early CNV in adolescents:

A continuous performance test (CPT) in the A-X version was employed. In this task, the cue ‘A’ was follwed with a probability of 50% by the target stimulus ‘X’ which required a speeded motor response. We analyzed different visual ERP components evoked by the cue ‘A’ from initial visual perception (P1, N2), to early and late visual CNV. Movement-related potentials have been addressed in a previous paper [22]. In analogy to our findings on movement-related potentials, we hypothesized that visual stimulus post-processing (early CNV) would be influenced more strongly than the initial stimulus processing stages related to visual perception (P1, N2 event-related potential components). More specifically, we hypothesized that occipito-temporal early CNV amplitudes would be genetically affected either by additive main effects of DAT1 and COMT as described in a working memory task [33] or that DAT1 and COMT would interact as demonstrated in the motor modality [22]. If we would find similar time-specific genetic modulation in both visual and motor post-processing, a supra-modal working memory encoding modulation process could account for these findings and would deserve further investigation.

## Methods

### Ethics statement

The study was approved by the institutional review board of the Medical Faculty of the University of Heidelberg/Mannheim. Written informed consent was obtained from all participants and their parents according to the Declaration of Helsinki.

### Participants

The exact sampling procedure has been described before [22]. In brief, from 195 healthy 15-year-olds (104 males, 91 females) of a community-based high-risk cohort [27], [34], 64-channel EEG data were obtained. Of the initial sample of 384 participants, 18 (4.7%) were excluded because of severe handicaps (neurological disorder, intelligence quotient <70 or motor quotient <70), 28 (7.3%) were drop-outs at age 15, 35 (9.1%) refused to take part in blood sampling or had incomplete genetic data, and from 43 (11.2%), no, or no reliable, EEG data were available. 65 subjects (16.7%) were excluded from the current analysis due to a current psychiatric DSM-IV diagnosis. All subjects were free of psychoactive medication at the time of the recording.

Hand dominance was assessed by the Edinburgh Handedness Inventory [35]. 174 (89.2%) subjects were right-handed, 14 were left-handed and 7 were ambidextrous. All subjects had a corrected visual acuity of 0.8 or higher. In contrast to the analysis of lateralized movement-related potentials [22], left handed subjects were not excluded in this paper because lateralization effects were not a focus of the current analysis.

### Recordings

Continuous 64-channel DC EEG was recorded by Neuroscan Synamps amplifiers (Neuroscan Inc., TX, USA). Sintered silver/silver chloride electrodes were positioned by an equidistant electrode cap (Easycap, FMS, Germany). Electrode impedances were kept below 10 kOhm. Vertical electrooculogram (VEOG) was recorded by electrodes 1 cm below and above the left eye. Horizontal electrooculogram (VEOG) was calculated by leads F9' and F10' next to the outer canthi. Small deviations of electrode positions from the international 10-10 system are indicated by apostrophes. The recording reference was placed near the left mastoid. Offline, data were transformed to average reference. The sampling rate was 500 Hz. An anti-aliasing low-pass filter with a cut-off frequency of 100 Hz was employed. The visual stimulation was presented by Gentask of the Neuroscan Stim software package. Reaction times were collected from response triggers from the response pad.

### Task

Subjects performed a computerized A-X version of the continuous performance test (CPT; constructed by doubling the number of trials of a common previous multicenter version [36], [37], [38]). 800 black-colored capital letters were presented on white background in the center of the computer screen for 150 ms. The stimulus onset asynchrony (SOA) between the different letters was 1600 ms. Whenever an ‘A’ was followed by an ‘X’ (50% probability), subjects had to respond with a fast right-hand button press with their index finger on the response pad. Correct responses within 1 second were accepted. The ‘A’ was followed by an ‘X’ 80 times and by another letter 80 times. Additionally, single distractor letters were presented.

An ‘X’ without a preceding ‘A’ occurred 80 times. Another nine letters of the alphabet (‘B’, ‘C’, ‘D’, ‘E’, ‘F’, ‘G’, ‘H’, ‘J’, ‘L’) were employed as distractors. The distractor ‘H’ occurred 160 times (frequent distractor). The distractors ‘B’, ‘C’, ‘D’, ‘E’, ‘F’, ‘G’, ‘J’, and ‘L’ appeared 40 times each.

### Data pre-processing

Average reference was calculated offline. Data were 30 Hz low-pass filtered by a zero-phase shift Butterworth filter with a slope of 48 dB/octave. Continuous recordings were segmented into epochs which comprised the time interval from 400 ms prior to the distractor before the warning stimulus ‘A’ to 1600 ms after the imperative stimulus ‘X’ (5.2 seconds in total). Data were corrected for eye movements and blinks by the algorithm of Gratton and Coles (Brain Vision Analyzer, BrainProducts GmbH, Munich, Germany). Potentials exceeding 150 µV amplitude were rejected automatically as artifacts; remaining smaller artifacts were removed by an experienced EEG technician who was blind to the study hypotheses.

### Data analysis

1. **Visual post-processing (occipito-temporal early CNV).** As the main target parameter, we analyzed visual post-processing after the warning stimulus ‘A’ (visual component of early CNV) during the interval 600–900 ms after stimulus onset, i.e. 450–750 ms after stimulus offset at the pooled leads P7', P8', P9', P10' [10], [12]. For comparison, the visual N700 following the imperative stimulus ‘X’ and following the distractors preceding the warning stimulus was examined at the same leads (P7', P8', P9', P10') and during the same time interval. Finally, the occipito-temporal early CNV was assessed in the same way in trials when the cue ‘A’ was followed by a non-target stimulus (though this does not allow to exclude trials with lapses of attention as no response was required in these trials).
2. **Visual perception and visual stimulus anticipation during late CNV.** In order to exclude that increased stimulus post-processing could be a by-product of increased anticipation or increased attention during the initial stimulus perception, we examined the same genetic effects on preceding visual potential components:
  - *Stimulus anticipation durin late CNV:* The parieto-occipital late contingent negative variation was determined over posterior parietal/dorsal occipital areas (P3', P4', PO1', PO2') during the 200 ms preceding the target stimulus [10], [39], [40].
  - *Perception/attentio:* P1 was calculated as the mean amplitude in a time window ±10 ms around the positive peak 70–140 ms after the warning stimulus at the pooled leads O1', O2', P7', P8' [12]. Visual N2 was calculated as the mean amplitude in a time window ±10 ms around the negative peak 140–230 ms after the warning stimulus at the pooled leads P7', P8', P9', P10'.
3. **Supramodal orienting response.** Finally, in order to complete the stimulus-locked analysis, genetic effects on orienting (frontal early CNV) were examined. Frontal early CNV amplitude was measured at its topographical maximum at Fz during the time interval 600–900 ms [41], [42].

### Source analysis of visual early CNV

Source analysis was carried out on the group grand averages which provide the best signal-to-noise ratio on time intervals which yielded significant genetic effects in the surface potential analysis [22]. Simplifying our previous dipole model [12], a 4-dipole model was fitted on the visual early CNV time interval (600–900 ms after the cue ‘A’) by the genetic algorithm implemented in BESA. 4 dipoles were chosen because the early CNV maps showed 3 negative peaks and a fourth dipole was introduced to check for additional activity. Residual variance and energy were minimized. Two dipoles were constrained to a symmetric location due to the symmetric occipito-temporal early CNV topography. As no systematic differences in visual early CNV potential topography or source localization were found between the genetic groups, the dipole model was fit on the *DAT1* 6R–10R/6R–10R+*COMT* Met/Met group (highest occipito-temporal early CNV amplitudes) and applied to other groups after refitting the dipole orientations.

In a second step sLORETA was performed in BESA research 5.3 (BESA GmbH, Fürstenfeldbruck, Germany) on the same early CNV time interval in order to describe the extension of the distributed cortical sources of visual early CNV as exact as possible. Default regularization parameters were employed assuring that neither over-regularization would lead to a low number of widespread midline sources nor an under-regularization to numerous superficial sources.

### Genotyping

EDTA anticoagulated venous blood samples were collected. Leukocyte genomic deoxyribonucleic acid (DNA) was isolated with the Qiamp DNA extraction kit (Qiagen, Chatsworth, California).

Genotyping of the *COMT* single nucleotide polymorphism (SNP) was completed using TaqMan (SNP) Genotyping Assays (7900HT Fast Real-Time-PCR-System; Applied Biosystems, Foster City, California). Amplification conditions for *COMT* rs4680 were: 3.0 µl TaqMan® Mastermix, 0.3 µl/0.15 µl TaqMan® oligonucleotide mix (20×/40×), 1.70 µl dH_2_O and 1 µl DNA solution (∼30 ng) in 96-well format in a 6 µl reaction. Amplification was performed by initial heating of 10 min–95°C, 40 cycles of 15 sec–95°C/1 min–60°C and final 10 min–4°C. TaqMan® assay-on-demand ID C_25746809_50 detected the alleles of rs4680 (hCV25746809) in the sequence context of CCAGCGGATGGTGGATTTCGCTGGC[A/G]TGAAGGACAAGGTGTGCATGCC.

The 40-bp VNTR polymorphism in 3′-untranslated-region (UTR) of *DAT1* was genotyped with the primers and reaction conditions of Sano et al. [43]. Polymerase chain reaction was carried out using a nucleotide mix consisting of 7.4 mM deoxyadenosine triphosphate, deoxycytidine triphosphate, and deoxythymidine triphosphate and 3.7 mM deoxyguanosine triphosphate and 7-deaza-2_-deoxyguanosine 5_-triphosphate (Amersham Biosciences, Piscataway, NJ). After an initial denaturation step, 35 cycles of amplification of 1 minute at 94°C, 1 minute at 63°C, and 35 seconds at 72°C were performed. The 30-bp intron 8 VNTR polymorphism was genotyped according to the procedure by Vandenbergh et al. [44]. All genotypes were scored independently by 2 individuals who were blind to the presented data. The VNTRs had been genotyped in the context of a previous study [27]. The following genotype distribution was obtained for the 30 bp *DAT1* VNTR in intron 8 (5R/5R N = 7; 5R/6R N = 62; 6R/6R N = 119; 5R/7R N = 2; 6R/7R N = 5) and the 40 bp *DAT1* VNTR at the 3′UTR (7R/9R N = 3; 9R/9R N = 11; 9R/10R N = 76; 10R/10R N = 104; 10R/11R N = 1). No deviations from Hardy Weinberg equilibrium were detected (*DAT1* 30 bp VNTR intron 8 p = 0.78; *DAT1* 40 bp VNTR 3′UTR p = 0.10; *COMT* p = 0.20).

Both *DAT1* VNTRs were analyzed combined as haplotype. In accordance with previous literature and to avoid small groups containing only a low number of subjects, with respect to the *DAT1* haplotype, subjects were dichotomized into homozygous carriers of the 6R–10R haplotype, which have previously been demonstrated to increase the risk of psychiatric disorders [27] and those who carried at least one non-risk haplotype.

In detail, the following genotype groups were formed: (1) *DAT1* haplotype: 6R–10R/6R–10R (N = 90) versus at least one non-6R–10R haplotype (N = 105); and (2) *COMT*: Val/Val (N = 43) versus Val/Met (N = 107) versus Met/Met (N = 45).

### Statistical analysis

To examine the effect of the *DAT1* haplotype (at least one non-6R–10R-haplotype was coded as ‘0’; 6R–10R/6R–10R was coded as ‘1’) and *COMT* (Val/Val = 0; Val/Met = 1; Met/Met = 2) on visual early CNV amplitude, linear regression analyses were performed. Gender was entered into the model as a covariate. In order to test for a significant epistasis between *DAT1* and *COMT*, regression models with and without an interaction term were compared. Significant interactions were further examined by univariate regression analyses with *COMT* alleles as predictor separately for each *DAT1* haplotype level, again controlling for gender effects.

We also tested whether the results would be confirmed when the visual N700 following the distractor and the visual N700 following the target stimulus ‘X’ were used as dependent variables, as we hypothesized that the visual N700 following any single visual stimulus could be the equivalent to early CNV following the cue ‘A’.

Finally, possible genetic effects on earlier stimulus processing and supramodal working memory updating/orienting were assessed by t-tests to examine the possibility that increased post-processing was only a by-product of increased earlier processing. No correction for multiple testing was applied in order to detect any confounding effects. Pearson correlation coefficients were computed to assess associations between occipito-temporal and frontal early CNV components as well as between initial visual perception-related components (P1, N2) and subsequent visual post-processing (occipito-temporal early CNV).

## Results

### Behavioral data

The task was well accomplished by the subjects, with on average 2.5±2.7 omission and 2.4±3.0 commission errors. Further details on mean reaction time, reaction time variability, omission and commission errors have been reported previously [22]. No significant genetic influences on reaction time or reaction time variability were observed. There was only a significant interaction between DAT1 and COMT polymorphisms with respect to omission errors (DAT1 haplotype beta = −0.25; t = 1.8; p = 0.07; COMT beta = −0.16; t = 1.7; p = 0.098; DAT1 haplotype×COMT beta = 0.31; t = 2.0; p = 0.047).

### Event-related potential data

#### Specificity of the time window: Effects of DAT1 and COMT on cue perception and target anticipation-related potential components

**Figure 1:**
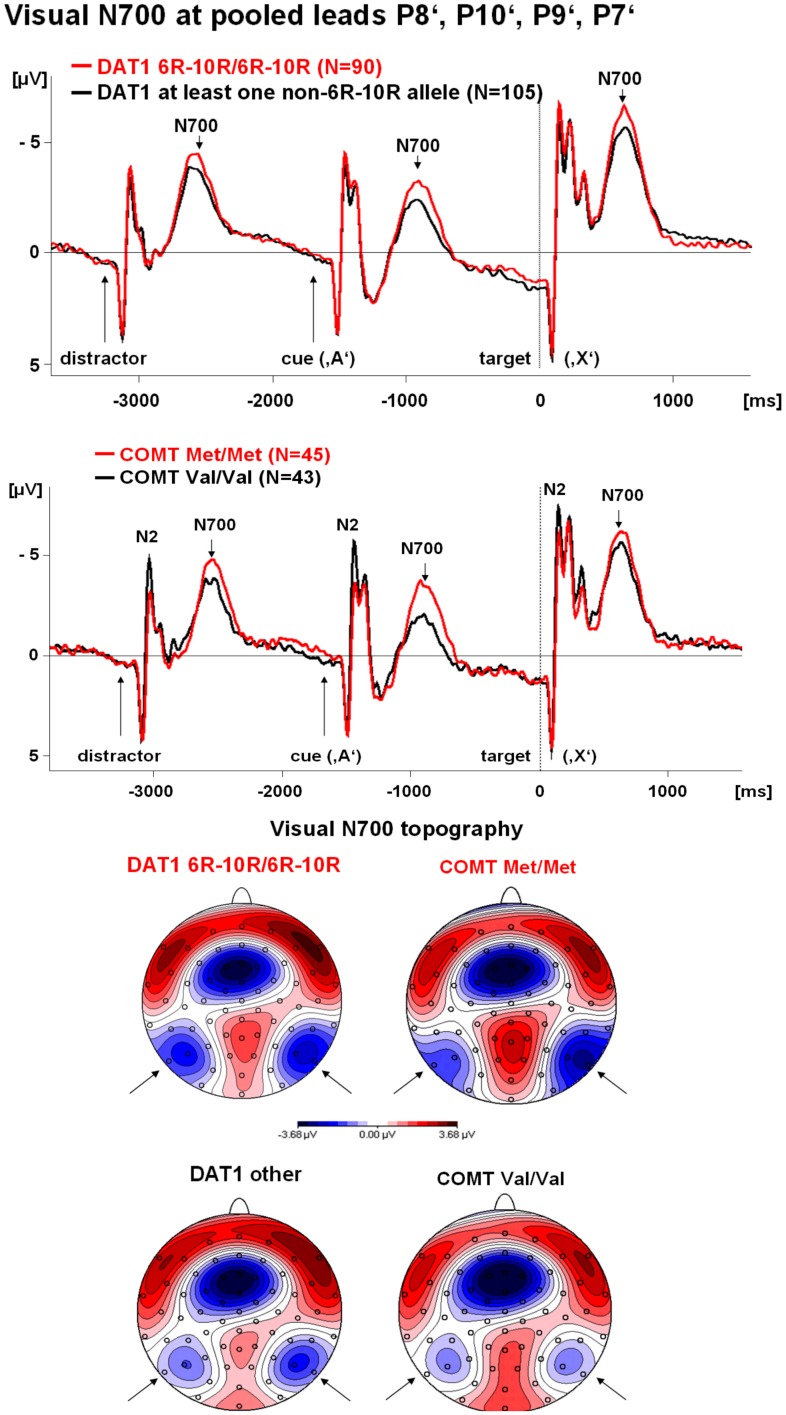
Time course and topography of the early visual contingent negative variation (CNV) by *DAT1* and *COMT* polymorphisms separately. **Top:** Time course by a) *DAT1* haplotype, and b) *COMT* Val^158^Met genotype. Negativity is indicated by upward deflections. Stimulus-locked averages are displayed, the vertical dashed line indicates the time of the button press. The visual post-processing interval is selectively and reliably affected. Only the *COMT* polymorphism seems to influence the visual N2 in the opposite direction to the visual early CNV. **Bottom:** Topography of the early CNV (600–900 ms after the cue ‘A’). Isopotential line maps are shown, negativity is illustrated by blue areas, positivity by red areas. The head is viewed from above, the nose is upwards. The small circles illustrate the position of the electrodes. Arrows mark the visual early CNV maximum. The topography appears to be unaltered by *DAT1* and *COMT* polymorphisms, but occipito-temporal early CNV amplitude is increased in the subjects with the Met/Met and the 6R–10R/6R–10R genotypes.

**Figure 2:**
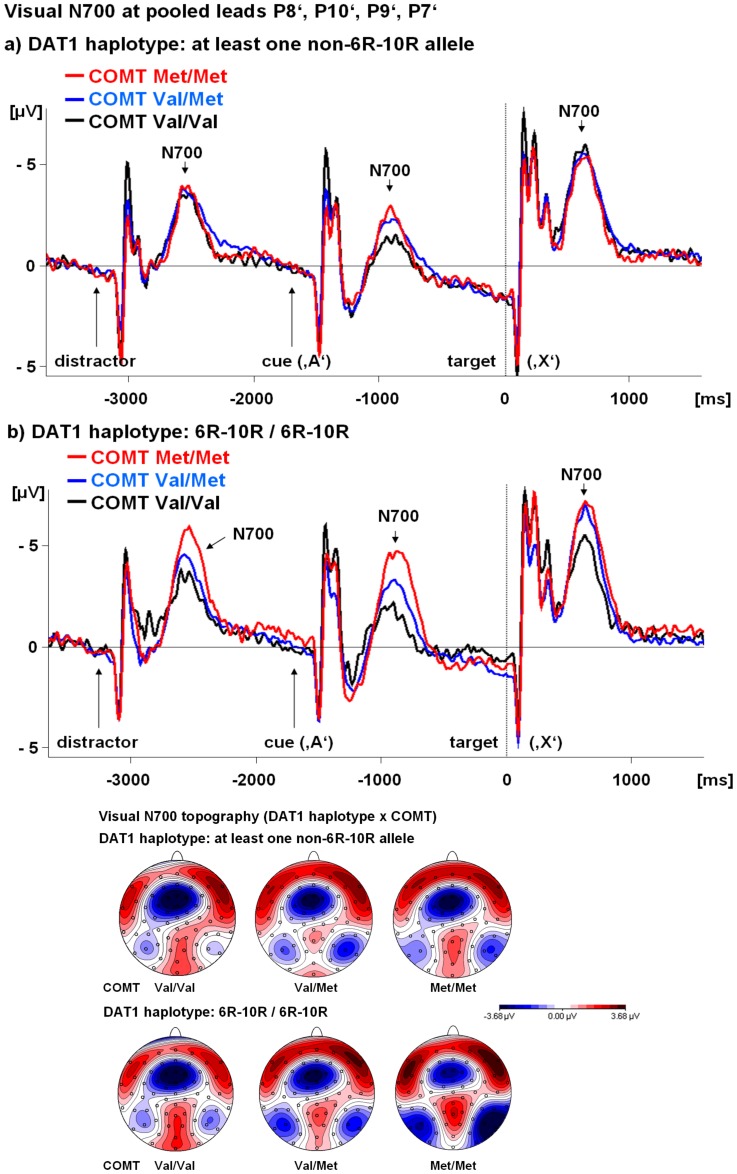
Combined effects of *DAT1* and *COMT* genotypes on early visual contingent negative variation (CNV). **Top:** The effect of the *COMT* genotype on the time course of the visual early CNV is shown separately for the homozygous 6R–10R *DAT1* haplotype and the *DAT1* haplotype with at least one non-6R–10R allele. The same conventions as for Figure 1 apply. **Bottom:** Topography of the visual early CNV (600–900 ms after the cue ‘A’) – combined influences of *DAT1* and *COMT* genotypes. Note that there were nearly no changes in visual early CNV topography but especially in the presence of the homozygous 6R–10R *DAT1* haplotype, the Met/Met *COMT* genotype increased visual occipito-temporal early CNV amplitude.

We obtained no significant effects of *DAT1* haplotype or *COMT* genotype on P1, N2, or occipito-parietal late CNV amplitude (all p>0.05). For any qualitative differences in P1, N2 or CNV between the genotype groups, see Figures 1 and 2. There was a trend towards a smaller visual N2 for the *COMT* Met/Met (−6.2±4.0 µV) versus the Val/Val group (−7.6±3.4 µV; t = 1.8; p = 0.08). Interactions between the *DAT1* haplotype and *COMT* genotype with respect to event-related potential components that preceded early visual CNV were far from being statistically significant (all p>0.46).

#### Early contingent negative variation following the warning stimulus ‘A’

Both *DAT1* and *COMT* polymorphisms had a significant effect on occipito-temporal early CNV amplitude (p = 0.02 and p = 0.05; linear regression model without an interaction term, see Tables 1 and 2). When an interaction term was included into the model, a trend towards an interaction between *DAT1* and *COMT* emerged (t = 1.8; p = 0.077).

**Table 1:** Means and standard deviations of visual early contingent negative variation (CNV) amplitudes by DAT1 and COMT.

Separate effects of DAT1 and COMT	visual early CNVamplitude [µV]‘A’	visual early CNVamplitude [µV]‘X’	visual early CNVamplitude [µV]‘other’
**DAT1 haplotype**			
6R–10R/6R–10R (N = 90)	−2.4±3.2 µV	−5.2±3.4 µV	−3.1±2.5 µV
Other (N = 105)	−1.7±2.3 µV	−4.6±2.8 µV	−2.6±2.0 µV
**COMT**			
Met/Met (N = 45)	−2.7±3.2 µV	−5.0±3.1 µV	−3.2±2.6 µV
Val/Met (N = 107)	−2.1±2.6 µV	−5.0±3.0 µV	−2.9±2.3 µV
Val/Val (N = 43)	−1.2±2.7 µV	−4.4±3.3 µV	−2.4±1.9 µV
**Combined effects of DAT1 and COMT**			
DAT1 10R–6R/10R-6R+COMT Met/Met (N = 20)	−3.8±3.8 µV	−5.7±3.7 µV	−4.1±3.0 µV
DAT1 other+COMT Met/Met (N = 25)	−1.8±2.3 µV	−4.3±2.4 µV	−2.5±2.1 µV
DAT1 10R-6R/10R-6R+COMT Val/Met (N = 53)	−2.3±2.9 µV	−5.3±3.4 µV	−2.9±2.5 µV
DAT1 other+COMT Val/Met (N = 54)	−1.8±2.2 µV	−4.7±2.6 µV	−2.8±2.1 µV
DAT1 10R-6R/10R-6R+COMT Val/Val (N = 17)	−1.2±2.9 µV	−4.1±2.9 µV	−2.4±1.9 µV
DAT1 other+COMT Val/Val (N = 26)	−1.3±2.6 µV	−4.6±3.6 µV	−2.5±1.9 µV

**Table 2:** Linear regression examining the effect of COMT and DAT1 haplotype on the visual early CNV following the cue ‘A’ (N = 195)^1^.

	B	SE B	beta	t-value	p
**without interaction (R^2^ = 0.06; p = 0.01)**					
Constant	−0.60±	0.89 µV			
Sex	−0.53±	0.39 µV	−0.10	1.4	n.s.
DAT1	−0.76±	0.39 µV	−0.14	1.94	0.05
COMT	−0.69±	0.29 µV	−0.17	2.4	0.02
					
**with interaction term DAT1×COMT (R^2^ = 0.07; p = 0.006)**					
Constant	−0.92±	1.23 µV			
Sex	−0.51±	0.39 µV	−0.09	1.3	n.s.
DAT1	0.30±	0.71 µV	0.05	0.4	n.s.
COMT	−0.25±	0.38 µV	−0.06	0.7	n.s.
DAT1×COMT	−1.04±	0.59 µV	−0.25	1.8	0.077

1 B = regression coefficient; SE B = standard error of the regression coefficient; beta = standardized regression coefficient.

Figure 1 illustrates how *DAT1* and *COMT* specifically affected the visual post-processing window following the distracter stimuli, the warning stimulus ‘A’ and the target stimulus ‘X’. The visual early CNV topography with the characteristic maximum over occipito-temporal visual areas is also presented. Figure 2 shows that the *COMT* effects tended to be stronger in the presence of the homozygous 6R–10R *DAT1* haplotype. There were no changes in visual early CNV topography or duration, but occipito-temporal early CNV *amplitude* was affected.

**Figure 3:**
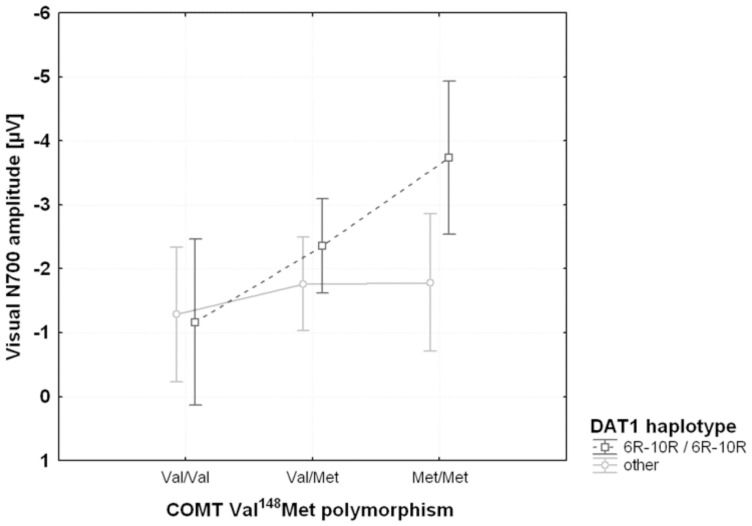
Effects of *DAT1* haplotype and *COMT* on visual early contingent negative variation (CNV). The error bars indicate the 95% confidence intervals.

Separate linear regression analyses for the two *DAT1*-haplotype-defined groups indicated that the *COMT* genotype only had a significant effect on visual early CNV amplitude in homozygous carriers of the 6R–10R *DAT1* haplotype (R^2^ = 0.07; regression slope −1.30±0.52 µV; beta = −0.26; t = −2.5; p = 0.01) but not otherwise (R^2^ = 0.03; regression slope −0.25±0.32 µV; beta = −0.08; t = −0.8; n.s.). The largest early visual CNV amplitudes occurred in those subjects carrying both the Met/Met *COMT* polymorphism and the homozygous 6R–10R *DAT1* haplotype (Figure 3).

The results were also roughly confirmed for the occipito-temporal early CNV followed by non-target stimuli in an additive model (R^2^ = 0.04; p = 0.03). *DAT1* regression slope was −0.67±0.40 µV (beta = −0.12; t = 1.7; p = 0.09) and *COMT* regression slope was −0.59±0.29 µV (beta = −0.14; t = 2.0; p = 0.046). Again, there was a trend towards an interaction of *DAT1* and *COMT* (t = 1.7; p = 0.099) in the model with an interaction term (R^2^ = 0.06; p = 0.01).

Accordingly, when all trials involving the cue ‘A’ (both followed by a target and by a non-target stimulus) were analyzed together, the additive effects of *COMT* (t = 2.3; p = 0.02) and *DAT1* (t = 1.9; p = 0.06) and the trend towards an interaction between *DAT1* and *COMT* (t = 1.8; p = 0.066) were confirmed.

**Figure 4:**
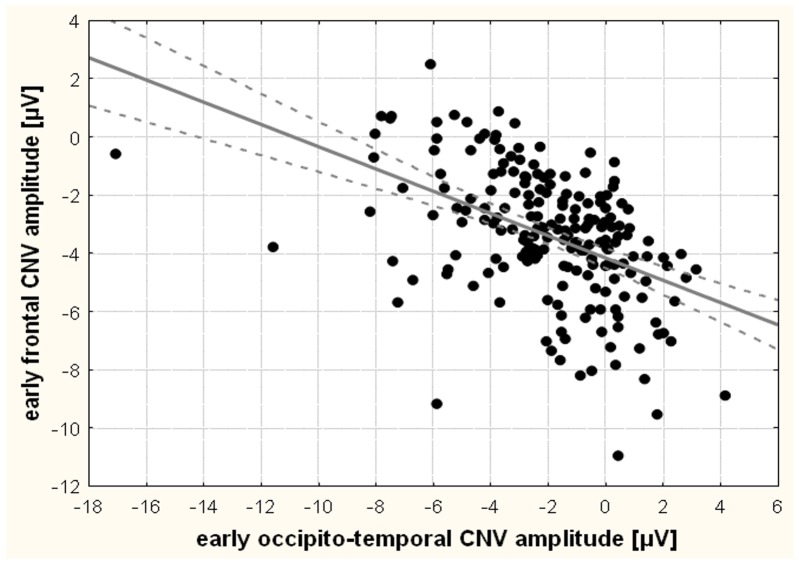
Scatterplot showing the negative correlation of frontal and occipito-temporal early contingent negative variation.

Though there was a trend towards an association of *COMT* genotype and early frontal CNV amplitude (beta = 0.12; t = 1.7; p = 0.09), there was no significant association of *DAT1* (beta = 0.03; t = 0.4; p = 0.69) or an interaction between *COMT* and *DAT1* (beta = 0.08; t = 0.5; p = 0.60) with respect to early frontal CNV. However, there was a strong negative correlation between early occipito-temporal and early frontal CNV (r = −0.47; t = 7.4; p<0.00001), i.e. larger negativity over visual areas was associated with *less* frontal negativity (Figure 4).

Moreover, there was a negative correlation of early occipito-temporal CNV with both omission errors (r = −0.22; t = 3.1; p = 0.003) and commission errors (r = −0.20; t = 2.8; p = 0.005) but not reaction time (r = −0.11; t = 1.5; p = 0.12), indicating that larger negativity during visual post-processing was associated with a higher number of both types of errors.

In contrast, there was no significant correlation between P1 (r = −0.08; t = 1.2; p = 0.25) or N2 amplitude (r = 0.07; t = 1.0; p = 0.33) and early occipito-temporal CNV amplitude despite the large sample size.

**Figure 5:**
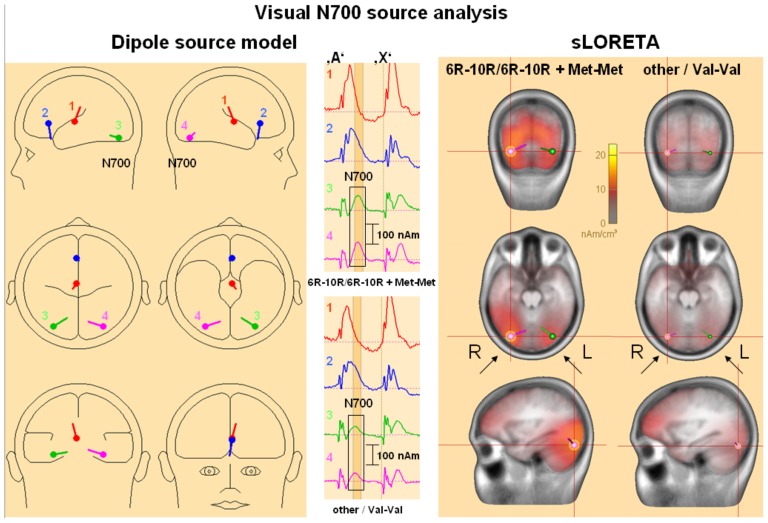
Dipole and sLORETA source analysis of influences of *COMT* and *DAT1* polymorphisms on early visual contingent negative variation (CNV). **Left:** 4-dipole source model fitted on the early CNV time interval by the genetic algorithm. Equivalent dipole 4 (pink) is symmetric to equivalent dipole 3 (green); both explain the occipito-temporal visual early CNV maxima. Dipoles 1 and 2 pick up additional frontal activity and activity related to the P300/late positive complex. **Middle:** Dipole moments for the homozygous 6R–10R/Met group (highest occipito-temporal early CNV amplitudes) compared to the homozygous other/Val group (low occipito-temporal early CNV amplitudes). Colours and numbers refer to the dipole model presented on the left, there were no qualitative differences between the groups. The vertical dashed line indicates the time of the imperative stimulus ‘X’, the early CNV time interval following the cue ‘A’ (600–900 ms) is marked in orange. **Right:** sLORETA source analysis for the same two genetic groups. The dipole locations of the dipole model depicted on the left are indicated. The occipito-temporal activation around Brodman areas 19, 37 (and adjacent areas) was stronger for the homozygous 6R–10R/Met group. The crossing red lines indicate the coordinates x = 0.40, y = −0.63, z = −0.14 (pink dipole).

***Source analysis:*** Residual variance during the visual early CNV time interval could be reduced to 7.7% by the 4-dipole model for the 6R–10R/6R–10R+Met/Met – group revealing the most pronounced visual early CNV. Figure 5 shows how two symmetrical occipito-temporal dipoles (dipole #3 and dipole #4) explained the visual early CNV time-course. Their dipole moments during the early CNV time interval were larger in the 6R–10R/6R–10R+Met/Met than in the *DAT1* other+*COMT* Val/Val group. For all groups, the dipole moments returned to baseline after the end of the visual early CNV component. sLORETA results pointed towards activation around Brodman areas 19 and 37. There were no hints towards topographic differences between the genetic groups. *DAT1* and *COMT* polymorphisms influenced the *extent* to which secondary visual areas became *transiently* and *selectively* active during visual post-processing.

#### Visual N700 following a preceding distractor and the target stimulus ‘X’/non-target stimuli which followed the cue ‘A’

Despite qualitative results for the visual N700 following the target stimulus ‘X’ which pointed in the same direction as for the visual N700 following the cue (Figures 1 and 2), the corresponding *DAT1* and *COMT* effects (DAT1 t = 1.4; p = 0.18; COMT t = 0.7; p = 0.46) and the interaction thereof (t = 1.4; p = 0.18) failed to reach trend or significance level in the linear regression model.

The corresponding additive *DAT1* and *COMT* effects on the visual N700 following non-target stimuli which had been preceded by the cue ‘A’ showed a trend for *DAT1* (t = 1.8; p = 0.07) but no effect of *COMT* (t = 0.7; p = 0.48), while the interaction between *DAT1* and *COMT* reached trend level in this case (t = 1.8; p = 0.08).

With respect to the visual N700 following the distracter which preceded the cue, there were also no significant effects of *DAT1* (t = 1.3; p = 0.19) or *COMT* (t = 1.5; p = 0.13) alone, but a trend towards an interaction between the two (t = 1.7; p = 0.08). Separate linear regression analyses for the two *DAT1*-haplotype-defined groups indicated that the *COMT* genotype had a significant effect on visual N700 amplitude in homozygous carriers of the 6R–10R *DAT1* haplotype only (R^2^ = 0.07; regression slope −0.85±0.41 µV; beta = −0.22; t = −2.1; p = 0.04) but not otherwise (R^2^ = 0.01 regression slope −0.01±0.28 µV; beta = −0.005; t = −0.05; n.s.). Again, the largest visual N700 amplitudes occurred in the subjects with the Met/Met *COMT* polymorphism in the presence of the homozygous 6R–10R *DAT1* haplotype.

## Discussion

The present investigation reports data on genomic imaging of the time course of visual processing. The findings outlined above provide first evidence that genetic variation in *DAT1* and *COMT* influence the amount of cortical activity during visual post-processing in the same specific time window about 500–1000 ms after the stimulus which we previously described for the motor system during movement post-processing [11], [22]. Most importantly, the post-processing time interval was again specifically affected, as there was no evidence for an increased processing during visual perception. To the contrary, there was even a trend towards less initial processing being accompanied by increased post-processing. Individuals with the Met/Met genotype showed higher early CNV amplitudes over visual or (contralateral motor areas [22]) than Val/Val carriers, while individuals with a heterozygous *COMT* exhibited intermediate occipito-temporal early CNV amplitudes. Individuals with the homozygous 6R–10R DAT1 haplotype showed higher visual early CNV amplitudes than subjects with at least one non-6R–10R *DAT1* allele. In agreement with Alexander's model [45], parallel fronto-striatal circuits could be modulated by *DAT1* and *COMT* with respect to visual and motor post-processing/working memory encoding.

There was a strong negative correlation between visual post-processing (occipito-temporal early CNV) and the orienting reaction as reflected by frontal early CNV, and both source analyses confirmed that these two negativities reflect distinct sources. Like in the motor system, where increased motor post-processing was associated with longer reaction times as an indicator for less attention and response preparation, again, visual post-processing was associated with a higher number of both omission and commission errors. I.e. worse task performance and less controlled attention were associated with increased post-processing. This could indicate that post-processing is used to compensate for deficits during initial stimulus perception.

However, frontal early CNV did not show the same or an inverse modulation by *DAT1* and *COMT* polymorphisms as did the occipito-temporal early CNV.

There was no significant correlation between ERP components during initial visual perception of the cue (P1, N2) and subsequent occipito-temporal early CNV. Taking into account the large sample size, this means that if initial visual perception would directly influence the amount of visual post-processing, any effect would have to be extremely small.

There was a trend towards an interaction between DAT1 and COMT for the visual early CNV following the cue ‘A’, the N700 following the preceding distractors and the non-X stimuli following the cue ‘A’ which required response inhibition. With respect to the N700 following the target stimuli, a ceiling effect may have been present, as overall higher amplitudes were obtained than during early CNV or N700 following a distractor. We suggest that the effects of *DAT1* and *COMT* on visual post-processing are not limited to paired stimulus paradigms (early CNV), but that a single visual stimulus is sufficient to evoke the same visual post-processing [11], [22]. The interaction effect between *DAT1* and *COMT* was not as clear as in the motor system [22]. Additive effects of *DAT1* and *COMT* have been described previously for the visual system in a working memory task [33].

As already observed in the motor system, the amplitude of visual early CNV/N700 was affected by *DAT1* and *COMT* and not N700 duration. This suggests that it was not the direct effect of *DAT1* or *COMT* on dopamine inactivation by reuptake or metabolism that was operative, but rather the effects of *DAT1* and *COMT* on tonic dopamine levels mediated the influences on visual post-processing [46]. The larger occipito-temporal early CNV amplitudes in carriers of the homozygous 6R–10R *DAT1* haplotype could also point towards visual stimuli eliciting larger phasic dopaminergic responses in the presence of lower tonic dopamine levels, taking into account that the 6R–10R *DAT1* haplotype has been associated with a higher dopamine turnover in children and adolescents [28]. The finding that our present analysis of the visual system yielded a similar time course as for the motor system could point towards the implication of subcortical key structures such as the basal ganglia [47] which are linked to widespread cortical areas.

Preceding visual event-related potential components during visual perception:

Overall, the results of the control conditions did not uphold the correction for multiple testing and must be examined in further studies. However, for the *COMT* Met/Met genotype among homozygous carriers of the 6R–10R *DAT1* haplotype, we obtained rather a combination of (non-significantly) *lower* initial processing followed by higher post-processing. Thus, no tentative influence of *COMT* and *DAT* genotypes pointed into the same direction as the influences of *COMT* and *DAT* on visual early CNV amplitude. There was no hint that the effects of *DAT1* and *COMT* on increased post-processing could have been a consequence of increased initial visual processing. *COMT* and *DAT1* genotype did not affect reaction time variability in this sample without subjects with attention deficit hyperactivity disorder [22]. However, this does not exclude the possibility that dopaminergic genes may interact with other factors with respect to reaction time variability.

Our results, thus, suggest several phases of dopaminergic effects on visual processing in visual event-related potentials and early CNV:

- Early visual processing: Dopamine may exert influences on early visual perception in the retina, though it is not an important neurotransmitter for the central pathways as shown in patients with Parkinsons's disease who have decreased visual P100 amplitudes [48]. A reduced P100 amplitude in schizophrenia has been found to be associated with working memory performance deficits [49]. However, *DAT1* and *COMT* polymorphisms did not affect early visual processing in our study. It remains unclear whether the association of reduced P100 in schizophrenia and working memory deficits may be an indirect effect due to a common source which affects both parameters.
- Early CNV and working memory encoding: Early CNV shows pronounced age effects during development with an amplitude decrease over frontal sites [42]. Developmental aspects may also change gene expression, as, in children, the 6R–10R-allele has been described to be a risk factor for ADHD, while in adults the 6R–9R allele was associated with ADHD [31], [32], pointing towards a differential decay of dopamine transporter expression with development [28], i.e. a steeper age-related decrease of dopamine binding capacity for non 6R–10R carriers. Previous findings with respect to CNV seem especially important for the interpretation of our results: Early frontal CNV is thought to represent an orienting reaction [7] which is strongly influenced by the modality of the cue [8]. CNV has been found to be increased by methylphenidate [50] though dopaminergic effects on CNV were not unanimously replicated [51]. However, in a widely accepted model, dopaminergic neurons in the basal ganglia can produce an inhibition of thalamic neurons which in turn disinhibits the cortex during CNV [9], [52], [53], [54]. Source analysis has yielded important generators of early CNV both in the frontal cortex (anterior cingulate cortex) as well as in the occipito-temporal visual cortex [10]. While frontal potentials have been associated with orienting and recruitment of resources for task performance [9], [10], modality-specific encoding in visual areas has been proposed to represent an important short-term memory buffer [5], [6]. Supramodal fronto-parietal circuits including supplementary motor area and the ACC have been identified [55] and could be related to our frontal negative component, as source analysis on grand average data can lead to a smearing of potentials and, thus, “deeper” equivalent source localizations. The negative correlation which we found between frontal early CNV and occipito-temporal early CNV could indicate that a well timed frontal resource allocation requires less processing in the occipito-temporal visual ventral stream to accomplish an effective working memory encoding.

### Conclusions

For the first time, dopaminergic genetic influences were demonstrated on the time course of visual processing in 15-year-old adolescents. We found that the same specific time interval as for the motor system was affected. We propose that modality-dependent memory traces in perceiving (visual) or executing (motor) systems are modulated during memory encoding by a dopaminergic system which affects the time interval of about 500–1000 ms after the stimulus or movement. Earlier perceptual stages were unaffected. Future studies will have to reveal the extent to which an inefficient resource allocation during the orienting response (reduced frontal early CNV associated with larger early CNV amplitudes over visual areas) affects performance, and contributes to an increased number of errors especially in adolescents.

## References

[1] Mishra J, Martinez A, Schroeder CE, Hillyard SA (2012). Spatial attention boosts short-latency neural responses in human visual cortex.. Neuroimage.

[2] Singhal A (2006). Differentiating between spatial and object-based working memory using complex stimuli: an erp study.. Int J Neurosci.

[3] Spencer KM, Dien J, Donchin E (2001). Spatiotemporal analysis of the late ERP responses to deviant stimuli.. Psychophysiology.

[4] Squires KC, Donchin E, Herning RI, McCarthy G (1977). On the influence of task relevance and stimulus probability on event-related-potential components.. Electroencephalogr Clin Neurophysiol.

[5] Ruchkin DS, Berndt RS, Johnson R, Ritter W, Grafman J (1997). Modality-specific processing streams in verbal working memory: evidence from spatio-temporal patterns of brain activity.. Brain Res Cogn Brain Res.

[6] Ruchkin DS, Grafman J, Cameron K, Berndt RS (2003). Working memory retention systems: a state of activated long-term memory.. Behav Brain Sci.

[7] Rohrbaugh JW, Syndulko K, Sanquist TF, Lindsley DB (1980). Synthesis of the contingent negative variation brain potential from noncontingent stimulus and motor elements.. Science.

[8] Gaillard AW (1976). Effects of warning-signal modality on the contingent negative variation (CNV).. Biol Psychol.

[9] Rockstroh B, Elbert T, Canavan A, Lutzenberger W, Birbaumer N (1989). Slow cortical potentials and behavior.

[10] Gomez CM, Marco J, Grau C (2003). Preparatory visuo-motor cortical network of the contingent negative variation estimated by current density.. Neuroimage.

[11] Bender S, Behringer S, Freitag CM, Resch F, Weisbrod M (2010). Transmodal comparison of auditory, motor, and visual post-processing with and without intentional short-term memory maintenance.. Clin Neurophysiol.

[12] Bender S, Oelkers-Ax R, Hellwig S, Resch F, Weisbrod M (2008). The topography of the scalp-recorded visual N700.. Clin Neurophysiol.

[13] McCollough AW, Machizawa MG, Vogel EK (2007). Electrophysiological measures of maintaining representations in visual working memory.. Cortex.

[14] Bender S, Becker D, Oelkers-Ax R, Weisbrod M (2006). Cortical motor areas are activated early in a characteristic sequence during post-movement processing.. Neuroimage.

[15] Bender S, Oelkers-Ax R, Resch F, Weisbrod M (2004). Motor processing after movement execution as revealed by evoked and induced activity.. Brain Res Cogn Brain Res.

[16] Bender S, Hellwig S, Resch F, Weisbrod M (2007). Am I safe? The ventrolateral prefrontal cortex ‘detects’ when an unpleasant event does not occur.. Neuroimage.

[17] Siniatchkin M, Kirsch E, Kropp P, Stephani U, Gerber WD (2000). Slow cortical potentials in migraine families.. Cephalalgia.

[18] Seamans JK, Yang CR (2004). The principal features and mechanisms of dopamine modulation in the prefrontal cortex.. Prog Neurobiol.

[19] Coull JT (1998). Neural correlates of attention and arousal: insights from electrophysiology, functional neuroimaging and psychopharmacology.. Prog Neurobiol.

[20] Karoum F, Chrapusta SJ, Egan MF (1994). 3-Methoxytyramine is the major metabolite of released dopamine in the rat frontal cortex: reassessment of the effects of antipsychotics on the dynamics of dopamine release and metabolism in the frontal cortex, nucleus accumbens, and striatum by a simple two pool model.. J Neurochem.

[21] Sesack SR, Hawrylak VA, Guido MA, Levey AI (1998). Cellular and subcellular localization of the dopamine transporter in rat cortex.. Adv Pharmacol.

[22] Bender S, Rellum T, Freitag C, Resch F, Rietschel M (in press). Dopamine Inactivation Efficacy related to Functional DAT1 and COMT Variants influences Motor Response Evaluation.. PLoS One.

[23] Lachman HM, Papolos DF, Saito T, Yu YM, Szumlanski CL (1996). Human catechol-O-methyltransferase pharmacogenetics: description of a functional polymorphism and its potential application to neuropsychiatric disorders.. Pharmacogenetics.

[24] Lotta T, Vidgren J, Tilgmann C, Ulmanen I, Melen K (1995). Kinetics of human soluble and membrane-bound catechol O-methyltransferase: a revised mechanism and description of the thermolabile variant of the enzyme.. Biochemistry.

[25] VanNess SH, Owens MJ, Kilts CD (2005). The variable number of tandem repeats element in DAT1 regulates in vitro dopamine transporter density.. BMC Genet.

[26] Brookes KJ, Neale BM, Sugden K, Khan N, Asherson P (2007). Relationship between VNTR polymorphisms of the human dopamine transporter gene and expression in post-mortem midbrain tissue.. Am J Med Genet B Neuropsychiatr Genet.

[27] Laucht M, Skowronek MH, Becker K, Schmidt MH, Esser G (2007). Interacting effects of the dopamine transporter gene and psychosocial adversity on attention-deficit/hyperactivity disorder symptoms among 15-year-olds from a high-risk community sample.. Arch Gen Psychiatry.

[28] Shumay E, Chen J, Fowler JS, Volkow ND (2011). Genotype and ancestry modulate brain's DAT availability in healthy humans.. PLoS One.

[29] Hahn T, Heinzel S, Dresler T, Plichta MM, Renner TJ (2011). Association between reward-related activation in the ventral striatum and trait reward sensitivity is moderated by dopamine transporter genotype.. Hum Brain Mapp.

[30] Dreher JC, Kohn P, Kolachana B, Weinberger DR, Berman KF (2009). Variation in dopamine genes influences responsivity of the human reward system.. Proc Natl Acad Sci U S A.

[31] Franke B, Hoogman M, Arias Vasquez A, Heister JG, Savelkoul PJ (2008). Association of the dopamine transporter (SLC6A3/DAT1) gene 9-6 haplotype with adult ADHD.. Am J Med Genet B Neuropsychiatr Genet.

[32] Franke B, Vasquez AA, Johansson S, Hoogman M, Romanos J (2010). Multicenter analysis of the SLC6A3/DAT1 VNTR haplotype in persistent ADHD suggests differential involvement of the gene in childhood and persistent ADHD.. Neuropsychopharmacology.

[33] Bertolino A, Blasi G, Latorre V, Rubino V, Rampino A (2006). Additive effects of genetic variation in dopamine regulating genes on working memory cortical activity in human brain.. J Neurosci.

[34] Laucht M, Esser G, Schmidt MH (1997). Developmental outcome of infants born with biological and psychosocial risks.. J Child Psychol Psychiatry.

[35] Oldfield RC (1971). The assessment and analysis of handedness: the Edinburgh inventory.. Neuropsychologia.

[36] van Leeuwen TH, Steinhausen HC, Overtoom CC, Pascual-Marqui RD, van't Klooster B (1998). The continuous performance test revisited with neuroelectric mapping: impaired orienting in children with attention deficits.. Behav Brain Res.

[37] Banaschewski T, Brandeis D, Heinrich H, Albrecht B, Brunner E (2003). Association of ADHD and conduct disorder–brain electrical evidence for the existence of a distinct subtype.. J Child Psychol Psychiatry.

[38] Brandeis D, Banaschewski T, Baving L, Georgiewa P, Blanz B (2002). Multicenter P300 brain mapping of impaired attention to cues in hyperkinetic children.. J Am Acad Child Adolesc Psychiatry.

[39] Bender S, Weisbrod M, Bornfleth H, Resch F, Oelkers-Ax R (2005). How do children prepare to react? Imaging maturation of motor preparation and stimulus anticipation by late contingent negative variation.. Neuroimage.

[40] Hellwig S, Weisbrod M, Jochum V, Rentrop M, Unger J (2008). Slow cortical potentials in human aversive trace conditioning.. Int J Psychophysiol.

[41] Bender S, Resch F, Weisbrod M, Oelkers-Ax R (2004). Specific task anticipation versus unspecific orienting reaction during early contingent negative variation.. Clin Neurophysiol.

[42] Bender S, Weisbrod M, Resch F, Oelkers-Ax R (2007). Stereotyped topography of different elevated contingent negative variation components in children with migraine without aura points towards a subcortical dysfunction.. Pain.

[43] Sano A, Kondoh K, Kakimoto Y, Kondo I (1993). A 40-nucleotide repeat polymorphism in the human dopamine transporter gene.. Hum Genet.

[44] Vandenbergh DJ, Thompson MD, Cook EH, Bendahhou E, Nguyen T (2000). Human dopamine transporter gene: coding region conservation among normal, Tourette's disorder, alcohol dependence and attention-deficit hyperactivity disorder populations.. Mol Psychiatry.

[45] Alexander GE, DeLong MR, Strick PL (1986). Parallel organization of functionally segregated circuits linking basal ganglia and cortex.. Annu Rev Neurosci.

[46] Owesson-White CA, Roitman MF, Sombers LA, Belle AM, Keithley RB (2012). Sources contributing to the average extracellular concentration of dopamine in the nucleus accumbens.. J Neurochem.

[47] Durston S, Fossella JA, Mulder MJ, Casey BJ, Ziermans TB (2008). Dopamine transporter genotype conveys familial risk of attention-deficit/hyperactivity disorder through striatal activation.. J Am Acad Child Adolesc Psychiatry.

[48] Nightingale S, Mitchell KW, Howe JW (1986). Visual evoked cortical potentials and pattern electroretinograms in Parkinson's disease and control subjects.. J Neurol Neurosurg Psychiatry.

[49] Koychev I, El-Deredy W, Haenschel C, Deakin JF (2010). Visual information processing deficits as biomarkers of vulnerability to schizophrenia: an event-related potential study in schizotypy.. Neuropsychologia.

[50] Linssen AM, Vuurman EF, Sambeth A, Nave S, Spooren W (2011). Contingent negative variation as a dopaminergic biomarker: evidence from dose-related effects of methylphenidate.. Psychopharmacology (Berl).

[51] Luthringer R, Rinaudo G, Toussaint M, Bailey P, Muller G (1999). Electroencephalographic characterization of brain dopaminergic stimulation by apomorphine in healthy volunteers.. Neuropsychobiology.

[52] Brunia CH (1993). Waiting in readiness: gating in attention and motor preparation.. Psychophysiology.

[53] Skinner JE, Yingling CD (1976). Regulation of slow potential shifts in nucleus reticularis thalami by the mesencephalic reticular formation and the frontal granular cortex.. Electroencephalogr Clin Neurophysiol.

[54] Yingling CD, Skinner JE (1976). Selective regulation of thalamic sensory relay nuclei by nucleus reticularis thalami.. Electroencephalogr Clin Neurophysiol.

[55] Gomez CM, Flores A, Ledesma A (2007). Fronto-parietal networks activation during the contingent negative variation period.. Brain Res Bull.

